# The New Horizon of Antipsychotics beyond the Classic Dopaminergic Hypothesis—The Case of the Xanomeline–Trospium Combination: A Systematic Review

**DOI:** 10.3390/ph17050610

**Published:** 2024-05-09

**Authors:** Octavian Vasiliu, Beatrice Budeanu, Mihai-Ștefan Cătănescu

**Affiliations:** 1 Department of Psychiatry, “Dr. Carol Davila” University Emergency Central Military Hospital, 010816 Bucharest, Romania; 2 Faculty of Medicine, « Carol Davila » University of Medicine and Pharmacy, 050474 Bucharest, Romania; beatrice.budeanu@yahoo.ro (B.B.); s.catanescu@yahoo.com (M.-Ș.C.)

**Keywords:** xanomeline, trospium, schizophrenia, Alzheimer’s disorder, psychoses, acetylcholine, muscarinic receptors, antipsychotics, dopaminergic hypothesis

## Abstract

Although the dopamine hypothesis of schizophrenia explains the effects of all the available antipsychotics in clinical use, there is an increasing need for developing new drugs for the treatment of the positive, negative, and cognitive symptoms of chronic psychoses. Xanomeline–trospium (KarXT) is a drug combination that is based on the essential role played by acetylcholine in the regulation of cognitive processes and the interactions between this neurotransmitter and other signaling pathways in the central nervous system, with a potential role in the onset of schizophrenia, Alzheimer’s disease, and substance use disorders. A systematic literature review that included four electronic databases (PubMed, Cochrane, Clarivate/Web of Science, and Google Scholar) and the US National Library of Medicine database for clinical trials detected twenty-one sources referring to fourteen studies focused on KarXT, out of which only four have available results. Based on the results of these trials, the short-term efficacy and tolerability of xanomeline–trospium are good, but more data are needed before this drug combination may be recommended for clinical use. However, on a theoretical level, the exploration of KarXT is useful for increasing the interest of researchers in finding new, non-dopaminergic, antipsychotics that could be used either as monotherapy or as add-on drugs.

## 1. Introduction

Schizophrenia spectrum disorders (SSDs) represent a constant challenge for clinicians, who are confronted with high rates of treatment resistance in their patients (with values as high as 34%), but also for researchers, who are facing the fact that there is no simple, single-neurotransmitter hypothesis of this disorder, and for caregivers, policymakers, and society, in its entirety, because the burden of schizophrenia worldwide, quantified by disability-adjusted life years (DALYs), is steadily increasing (by 65% from 1990 to 2019) [1,2,3,4]. In addition, the incidence and prevalence of schizophrenia increased worldwide by 37% and 65%, respectively, in the last two decades [1], suggesting the urgent need for finding adequate therapeutic intervention for this population.

The situation of SSDs is not an isolated case in Psychiatry because other severe mental disorders (SMDs), like bipolar disorders (BDs) or major depressive disorder (MDD), are also associated with high rates of treatment resistance, up to 25% for BDs (maybe underestimated due to lack of consensus for the criteria of non-responsivity) and 33% in patients with MDD (but again, the percentage is dependent on the criteria used) [5,6,7,8,9]. This type of treatment resistance is associated with lower quality of life, persistent functional impairment and productivity loss, and higher costs of healthcare in patients with BD or MDD [5,6].

Antipsychotics are the mainstay in the treatment of SSDs and have an important role in other SMDs, either as first-line agents (e.g., atypical antipsychotics in manic episodes) or as add-on agents (e.g., in MDD with psychotic features or in neurocognitive disorders with episodic delusions and/or hallucinations) [10,11,12,13,14,15]. All the available antipsychotics for clinical use, including pimavanserin, which was recently approved by the U.S. Food and Drug Administration (FDA) only for the management of hallucinations and delusions associated with Parkinson’s disease, are based on the dopaminergic hypothesis of psychosis [16,17,18,19,20,21]. Despite more than 60 years of research, only one medication, clozapine, has been approved for treatment-resistant cases of schizophrenia (TRS) [22,23,24]. Additionally, there are some patients who do not respond to clozapine and are classified as clozapine-resistant or “ultra-treatment-resistant” cases [25]. When taking this aspect into consideration and adding the low tolerability and the severe risks of safety related to clozapine, it becomes evident that more therapeutical options are needed for patients with TRS.

The dopamine hypothesis of psychosis stands as one of the most enduring theories in the field of psychopharmacology, and although it was constantly amended [14,15,26], no other pathogenetic hypothesis can yet be supported with the same level of confidence. The predominant version of this hypothesis involves the existence of reduced dopaminergic activity in the prefrontal cerebral region (explaining the onset and maintenance of negative symptoms in schizophrenia) juxtaposed with heightened dopaminergic activity in the limbic system (responsible for the pathogenesis of positive symptoms in SSDs) [27]. The varying levels of dopaminergic activity are attributed to receptor distribution, particularly the dopamine D1 and D2, in frontal and limbic regions, respectively, as well as the integrity of dopaminergic circuits, such as the mesocortical and mesolimbic pathways [28]. 

However, in the last decade, other pathophysiological mechanisms have been investigated in SSDs, and new drugs are in the pipeline that explore different neurotransmitter pathways [29,30]. For example, dysfunctions of serotonin, glutamate, gamma-aminobutyric acid (GABA), acetylcholine (Ach), norepinephrine, or cannabinoid systems, but also other non-neurotransmitter-related mechanisms, like dysregulation of gene expression, alteration of immune reactions, or neurodevelopmental abnormalities, have all been suggested as potential contributors to the onset of SSDs [29,30,31,32]. Also, the modulation of the trace amine-associated receptor 1 (TAAR-1) is being investigated as a possible way to reduce aberrant dopamine signaling and improve cerebral metabolism [33,34].

Acetylcholine plays a crucial role as a neurotransmitter in the body and brain, and its impact on muscarinic receptors (mAch) holds particular significance in SSDs [35,36,37,38]. The importance of cholinergic transmission in the central nervous system (CNS) derives from the ability of this neurotransmitter to regulate dopaminergic, GABA-ergic, and glutamatergic signaling [39,40], making the modulation of Ach receptors a promising focus for many neurological and psychiatric illnesses. The five human muscarinic acetylcholine receptors (M1–M5 mAChRs) belong to Class A of the G-protein-coupled receptors (GPCRs) and are extensively distributed in both the central and peripheral nervous systems [41,42]. These receptors play essential roles in regulating cognition and the development of psychosis and addiction, making them important targets for treating various CNS disorders such as Alzheimer’s disease (AD), schizophrenia, and drug dependence [42]. Primarily, type 4 mACh has been located in different areas of the CNS and is associated with cognitive functioning, addictive behaviors, and psychotic symptoms [43]. While M1 receptors stimulate dopamine release via dopaminergic afferents, M2 and M4 receptors function as autoreceptors, regulating Ach release from cholinergic interneurons [44]. 

Muscarinic ACh receptors, particularly the M1, M4, and M5 subtypes, hold potential as targets for innovative treatments addressing schizophrenia-related circuitry modulation [45]. There is significant evidence that the cholinergic system modulates the striatal dopaminergic signal by activating mAChRs and nicotine ACh receptors (nAChRs) through cholinergic projections from the brain to the middle brain, stimulating dopamine neurons in the substantia nigra and ventral tegmental area [46]. Studies have revealed a decrease in mAch receptors in chronically medicated individuals with schizophrenia, alongside reduced cholinergic cell bodies in the nucleus basalis of Meynert [47]. The involvement of M4 receptors in regulating dopamine neurotransmission in the striatum is important for explaining why M4-positive allosteric modulators have a therapeutic effect without antagonizing D2 receptors in the brain, as is the case for antipsychotic agents [45]. Variations in muscarinic M1 receptors in the dorsolateral prefrontal cortex are related to cognitive (primarily verbal learning and memory) and negative symptoms in psychotic disorders [48]. In preclinical models, positive allosteric modulators of M1 and M4 receptors can mediate pro-cognitive and antipsychotic-like activity, while negative allosteric modulators selectively targeting M5 receptors have been associated with favorable effects on negative, positive, and cognitive symptoms [45]. This intricate interplay underscores the importance of muscarinic effects in modulating dopamine transmission, offering insights into the pathophysiology of schizophrenia.

In the context of the urgent need to find therapeutic solutions for SSDs, the combination of xanomeline (3-(4-hexoxy-1,2,5-thiadiazol-3-yl)-1-methyl-5,6-dihydro-2H-pyridine), an agonist of the muscarinic M1 and M4 receptors, and trospium chloride (3-(2-hydroxy-2,2-diphenylacetoxy)spiro[bicyclo [3.2.1]octane-8,1′-pyrrolidin]-1′-ium chloride), a peripheral cholinergic antagonist, is explored for the treatment of schizophrenia [43,49,50,51,52]. In vitro and in vivo studies suggest xanomeline is a dual orthosteric and allosteric ligand at the human M4 cholinergic receptors, highlighting the complex pharmacodynamic profile of this pharmacological agent [43]. Xanomeline is also a strong M5 partial agonist/antagonist, a potent agonist of serotonergic 5HT1A and 5HT1B receptors, and an antagonist of the 5HT2 receptors [53]. It can pass through the hematoencephalic membrane because of its highly polar structure [54]. Preclinical evidence shows that in M4 mAChR knockout (KO) mice, xanomeline’s antipsychotic effects were entirely nullified, whereas in M1 mAChR KO mice, its impact was only partially diminished [55]. According to this study’s results, xanomeline can simultaneously bind to a single M4 mAChR at two sites: the classic orthosteric site and an extracellular vestibule allosteric site [55]. This represents the first instance where a small-molecule clinical drug candidate exhibits such simultaneous binding modes on a single GPCR. These findings have significant implications for understanding GPCR modulation and using structural biology in drug discovery. Moreover, this sheds new light on how xanomeline functions in treating schizophrenia, offering insights into its atypical pharmacology [43,56]. Although initially explored, as well as other selective muscarinic receptor agonists, for the treatment of AD, xanomeline was not approved for this indication due to its poor tolerability despite its positive effects on cognitive function [50,51].

First being developed for overactive bladder, trospium is a cholinergic antagonist in peripheral receptors. In theory, the combination of xanomeline and trospium should confer better acceptability with an elevated safety profile by decreasing the impact of cholinergic receptor activation induced by xanomeline in the periphery. Trospium is approved for treating overactive bladder in the US and European Union [57,58].

The US Food and Drug Administration (FDA) has accepted the New Drug Application (NDA) for KarXT (xanomeline–trospium) to treat schizophrenia in adults [59]. The application has been assigned a Prescription Drug User Fee Act (PDUFA) date of 26 September 2024 [60,61].

This review is focused on the evaluation of xanomeline–trospium efficacy and tolerability, and its usefulness is granted by (1) the need to find new therapeutic resources for SMDs, and especially for SSDs, that may reduce the rate of non-responsiveness; (2) this combination of drugs is supposed to avoid the onset of the adverse events associated with already marketed antipsychotics, due to its different mechanism of action; and (3) the potential implications of this drugs association to advance the knowledge on the pathophysiology of SSDs.

## 2. Methods

A systematic review was conducted to analyze studies on xanomeline–trospium association across various stages of clinical research. The review encompassed four electronic databases, i.e., PubMed—https://pubmed.ncbi.nlm.nih.gov/ accessed on 1 march 2024; Cochrane Database of Systematic Reviews—https://www.cochranelibrary.com/ accessed on 1 March 2024; Clarivate/Web of Science—https://www.webofscience.com accessed on 1 March 2024; and Google Scholar—https://scholar.google.com/ accessed on 1 March 2024. The search query utilized the terms “KarXT” OR “xanomeline-trospium” AND “clinical trial” OR “clinical study” OR “efficacy” OR “tolerability” OR “safety” AND “schizophrenia” OR “schizophrenia spectrum disorders” OR “severe mental disorders”. Additionally, references from articles were scrutinized and added to verify if they were not captured in the initial search. Also, due to the topic’s novelty, the grey literature was searched, including the manufacturer’s website and other websites dedicated to press releases about drugs in the pipeline. A supplementary search targeted ongoing trials of xanomeline–trospium listed in the US National Library of Medicine (https://clinicaltrials.gov/ accessed on 1 March 2024).

The primary search included all relevant papers and references from the electronic databases published from the respective databases’ inception until March 2024, written in English.

This systematic review adheres to the Preferred Reporting Items for Systematic Reviews and Meta-Analyses (PRISMA) 2020 statement [62] (Supplementary Materials, Table S1). The inclusion and exclusion criteria were formulated according to the PICO (patient, intervention, comparison, outcome) algorithm [63] and are listed in Table 1. The risk of bias was assessed using the GRADE recommendations (Table 2) [64]. The overall quality of data assessment was based on GRADE recommendations for clinical trials, according to which there are five elements that rate down their quality (i.e., risk of bias, inconsistency, indirectness, imprecision, and publication bias) and three rating-up elements (i.e., large effect, dose–response effect, and effect of all plausible confounders) [64]. Two authors independently rated the quality of data for each source (from “very low” to “high”), and, in case of disagreement, the third author mediated the consensus (Supplementary Materials, Table S2).

## 3. Results

**Figure 1:**
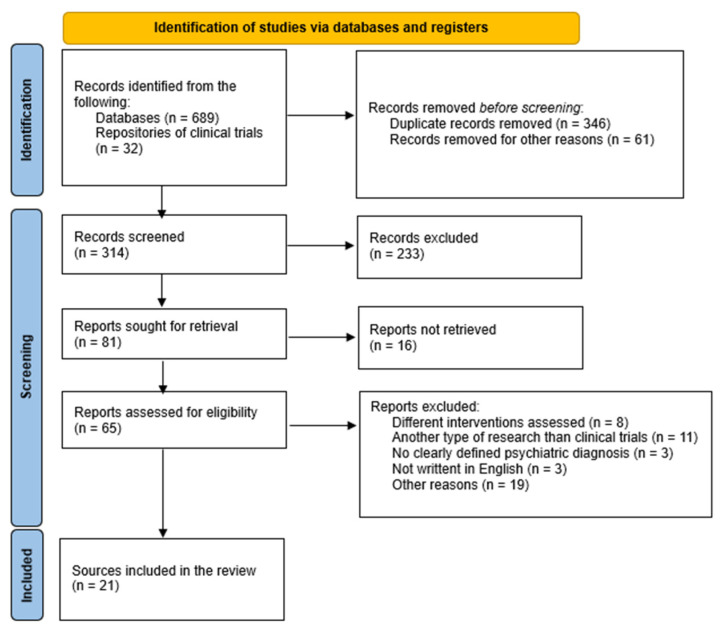
Results of the PRISMA-based search paradigm [62].

After the literature search and application of inclusion/exclusion criteria, 21 sources referring to 14 clinical trials with xanomeline–trospium were identified (Figure 1). Data about the design of the study and main results (where available) are presented synthetically in Table 3 and Table 4. Of the retrieved clinical trials, six were finalized, out of which only four have available results, four trials were ongoing, and the other four were planned to begin in the near future. Out of the six finalized trials identified, three were of Phase III, two of Phase I, and one of Phase II, while the other eight (ongoing/planned to begin) were of Phase III.

### 3.1. Efficacy of Xanomeline–Trospium

The primary outcome included in the reviewed trials was the change in the Positive and Negative Syndrome Scale (PANSS) and/or Brief Psychiatric Rating Scale (BPRS) total score. These instruments have good validity and reliability, as demonstrated by a large number of clinical studies [68,69,70]. Three trials (a phase II and two phase III) are reviewed in this section because they contained the efficacy of the xanomeline–trospium combination in their outcomes.

A phase 2, double-blind, placebo-controlled trial (EMERGENT-1) enrolled 182 inpatients diagnosed with schizophrenia who were in an acute exacerbation phase and randomized them on KarXT (doses titrated up to 125/30 mg b.i.d, flexible regimen) vs. placebo [65,71]. The main outcome was the change in PANSS total scores from baseline to week 5, and the secondary outcomes were changes in PANSS positive and negative scores, Clinical Global Impression Severity (CGI-S) scores, PANSS Marder negative score, and percentage of responders on CGI-S (scores equal to 1 or 2) [65]. In a paper containing the results of this trial, the PANSS total score decreased significantly in the KarXT-treated patients vs. those with placebo (−17.4 vs. −5.9 at week 5, *p* < 0.001) [72]. Also, the secondary outcomes reflected a significant improvement in the xanomeline–trospium group vs. placebo, except for the percentage of patients with a CGI-S response [72]. A post hoc analysis of data resulting from this trial (N = 83 patients treated with KarXT and 87 with placebo) showed a response rate between 59% (defined by ≥20% PANSS total scores reduction between baseline and endpoint) and 15.7% (≥50% PANSS reduction) [73]. All response rates were significantly superior in patients who received KarXT vs. placebo, and the number-needed-to-treat (NNT) values were between 3 (≥20% improvement) and 11 (≥50% improvement) [73]. KarXT led to a significantly superior response vs. placebo as early as two weeks (≥20% and ≥30% improvement) and 4 weeks (≥40% and ≥50% improvement) [73]. Also, the five Marder factors on PANSS (positive, negative, disorganized thought, hostility, and anxiety/depression) reflected a significant difference between the active drug and placebo starting from week 2 up to the end of the trial (week 5) [73]. Sauder et al. [74] underline, in another post hoc analysis of the EMERGENT-1 trial results, that a tendency towards more significant enhancement in cognitive function was observed with KarXT compared to the placebo throughout the 5-week treatment period, although statistical significance was not attained (mean included PANSS 96.0  ±  7.6, mean excluded PANSS 97.5  ±  13.2, *p*  =  0.60).

A phase 3, randomized, double-blind, parallel-group, placebo-controlled inpatient study (EMERGENT-2) explored the efficacy and safety of KarXT in adults with acute psychosis and a diagnosis of schizophrenia [57,75]. The primary objective was the change in PANSS total scores during the administration of xanomeline–trospium (125/30 mg b.i.d) for five weeks, and the enrolled disclosed number of participants was 252 [57]. The results available on the manufacturer’s site (but not published in any journal) support the efficacy of KarXT vs. the placebo on the PANSS negative subscore and Marder negative score. There was a notable reduction in the PANSS total score when comparing KarXT (baseline 98.3) to the placebo (baseline 97.9) after five weeks, with scores of 77.1 and 86.3, respectively (*p* < 0.0001) [75].

Yet another phase 3 trial, EMERGENT-3, with a randomized, double-blind, placebo-controlled design, enrolled 256 patients with schizophrenia and explored the efficacy of KarXT (125/30 mg b.i.d) vs. placebo in reducing the PANSS total scores during five weeks [76]. As secondary objectives, this study intended to evaluate the improvement in symptom severity, changes in safety and tolerability, and pharmacokinetics in the study group [76]. The study appears to be completed as of December 2022 [76], but no results have yet been posted.

### 3.2. Tolerability and Safety of Xanomeline–Trospium

The data presented in this section are derived from five clinical trials that describe the tolerability and safety of the xanomeline–trospium combination within their outcomes or results.

Based on the analysis of the previously mentioned studies (EMERGENT-1 and EMERGENT-2 trials), adverse events due to peripheric muscarinic receptor agonism, such as nausea, vomiting, diarrhea, sweating, and hypersalivation, have been reported, raising significant tolerability challenges for case management [57,72]. The majority of cholinergic adverse events with KarXT were mild, had an early onset (i.e., the first 1−2 weeks), and were transient (1–13 days) [71]. KarXT was associated with no significant changes in body weight, metabolic parameters, somnolence, or vital signs vs. the placebo [71]. All pro-cholinergic AEs in the KarXT group were transient, especially vomiting, which resolved within 1 day in most cases and had a maximum duration of 3 days [71]. The median duration of nausea was 9 days with KarXT and 15 days with the placebo [71]. Anticholinergic adverse events with KarXT lasted a median duration of 13 days for dry mouth and 5 days for constipation compared with 7 days and 17 days, respectively, with the placebo [71]. The NNH values for KarXT, derived from the results of this trial, were 9 for nausea, 23 for vomiting, 8 for constipation, and 13 for dry mouth [71].

According to the results of a phase I, double-blind, randomized, multiple-dose pilot study, adding trospium to xanomeline led to a 50% decrease in the incidence of muscarinic adverse events compared to xanomeline as monotherapy [77,78]. This inpatient study enrolled 70 healthy volunteers who were randomized to xanomeline + trospium (225 mg/day + 40 mg/day) or xanomeline/placebo (225 mg/day), and the duration of monitoring was seven days [77,78]. The proportion of subjects reporting any treatment-emergent adverse events (TEAEs) was 81.8% on xanomeline alone and 65.7% on KarXT [77,78]. There was a 46% reduction in the incidence of any cholinergic adverse events reported by subjects treated with KarXT compared with xanomeline alone (34.3% vs. 63.6%, respectively), and KarXT was associated with a 59% reduction in sweating [77,78]. In addition, there was a reduction of ≥29% in the incidence of each of the four other individual cholinergic adverse events by KarXT compared with xanomeline alone [77,78]. ECGs, vital signs, and laboratory values were similar between the treatment arms. There were no episodes of syncope in KarXT-treated subjects (two cases occurred in the xanomeline-alone arm), and postural dizziness was noted at lower rates in the KarXT arm (11.4%) compared with xanomeline alone (27.2%) [77,78].

Another phase I trial enrolled 69 healthy volunteers and included a 2-day titration period of either placebo or a KarXT dose of 50 mg xanomeline + 20 mg trospium followed by a 5-day treatment period (xanomeline 100 mg, 125 mg, and 150 mg in combination with trospium 20 mg or 40 mg) [66]. Most cholinergic adverse events occurred within the first few days of starting or increasing the study drug [66]. The majority of these adverse events appeared at 100 mg and 125 mg xanomeline-dose levels but were mild and transient in nature; none of the study groups showed meaningful changes in orthostatic heart rate or obvious differences in blood pressure between the placebo and KarXT [66]. Increasing the trospium dose ameliorated cholinergic adverse events and led to the observance of some anticholinergic adverse events [66]. Some cohorts tested on 40 mg trospium b.i.d reported signs of anticholinergic effects (i.e., dry mouth), particularly in the cohort receiving 125 mg b.i.d. of xanomeline [66].

A phase 3b open-label trial, with a duration of three years, had as a target of enrollment 380 patients with schizophrenia who did not tolerate their ongoing medication or did not have enough controlled symptoms, and the main objective was to assess the long-term safety, tolerability, effectiveness, and durability of the effect of KarXT [79]. The primary outcome of this trial was the incidence of TEAEs leading to discontinuation and the effectiveness of KarXT, reflected in the total Investigator Assessment Questionnaire (IAQ) and CGI-S scores [79]. The doses of KarXT used were 50/20 mg b.i.d. to 125/30 mg b.i.d. [79]. The study appears to be completed as of March 2023, but no results were posted [79].

**Table 3:** Efficacy and tolerability of KarXT based on finalized clinical trials.

Reference	Clinical trial Phase and Registration Number	Design	Results	OQD
Karuna Therap. [65], Correll et al. [71], Brannan SK [72], Weiden PJ [73], Sauder C [74]	Phase 2, NCT03697252 (EMERGENT-1)	DBRCT, N = 182 adult inpatients, acute schizophrenia. The KarXT (mg xanomeline/mg trospium) dosing schedule was flexible, starting with 50 mg/20 mg b.i.d and increasing to a maximum of 125 mg/30 mg b.i.d. Main outcome: PANSS-T score at week 5. Secondary outcomes: PANSS-P, PANSS-N, and PANSS-M-N scores, CGI-S, and % of responders (CGI-S) at week 5.	PANSS-T score ↓ significantly in KarXT-treated patients vs. placebo (−17.4 vs. −5.9 at week 5, *p* < 0.001). Secondary outcomes—significant improvement in the active group vs. placebo, except for the % CGI-S responders. Response rates between 15.7 and 59% (defined by >20–50% ↓PANSS-T scores). The five Marder factors on PANSS showed a significant difference between the active drug and placebo from week 2 to the end of the trial. A tendency towards more significant enhancement in cognitive function with KarXT compared to placebo was reported. The most common AEs (occurring in ≥2% of patients in the KarXT group and at a more than two-fold higher incidence than in the placebo group) were nausea (16.9% vs. 4.4%), vomiting (9.0% vs. 4.4%), constipation (16.9% vs. 3.3%), and dry mouth (9.0% vs. 1.1%).	High
Karuna Therap. [57], Kaul et al., 2023 [75]	Phase 3 trial, NCT04659161 (EMERGENT-2)	DBRCT, N = 252 adult inpatients with acute schizophrenia. KarXT was administered for 5 weeks: 50 mg xanomeline and 20 mg trospium b.i.d for the first 2 days + 100 mg xanomeline and 20 mg trospium b.i.d for days 3–7 + on day 8, KarXT dosing was flexible with an optional increase to 125 mg xanomeline and 30 mg trospium b.i.d and the option to return to 100 mg xanomeline and 20 mg trospium based on tolerability. Main outcome: PANSS-T score at week 5. Secondary outcomes: PANSS-P, PANSS-N, PANSS-M-N, CGI-S scores at week 5, and % of responders (CGI-S).	PANSS scores decreased significantly (*p* < 0.0001) at endpoint vs. placebo. The most common AEs with KarXT vs. placebo were constipation (27 [21%] vs. 13 [10%]), dyspepsia (24 [19%] vs. 10 [8%]), headache (17 [14%] vs. 15 [12%]), nausea (24 [19%] vs. 7 [6%]), vomiting (18 [14%] vs. 1 [1%]), hypertension (12 [10%] vs. 1 [1%]), dizziness (11 [9%] vs. 4 [3%]), gastro-esophageal reflux disease (8 [6%] vs. 0 [0%]), and diarrhea (7 [6%] vs. 4 [3%]). TEAEs rates of extrapyramidal motor symptoms (KarXT, zero [0%] vs. placebo, zero [0%]), akathisia (one [1%] vs. one [1%]), weight gain (zero [0%] vs. one [1%]), and somnolence (six [5%] vs. five [4%]).	High
Karuna Therap. [76]	Phase 3, NCT04738123 (EMERGENT-3)	DBRCT, N = 256 adult inpatients with schizophrenia, KarXT (125 mg xanomeline/30 mg trospium b.i.d) vs. placebo. Main outcome: PANSS-T score at week 5. Secondary outcomes: PANSS-P, PANSS-N, PANSS-M-N, CGI-S scores at week 5, and % of responders (PANSS-T).	No results posted.	N/A
Karuna Therap. [79]	Phase 3b, NCT05643170 (PENNANT)	OL, N = 380 (estimated), 4 (actual enrollment) patients with schizophrenia who did not tolerate/respond to current medication, KarXT 50/20 mg b.i.d, 100/20 mg b.i.d., or 125/30 mg b.i.d, 3 years. Main outcome: TEAEs leading to discontinuation; persistence and durability of KarXT effect (IAQ and CGI-S scores). Secondary outcomes: TEAEs incidence, CGI-I, and MSQ scores.	Not released.	N/A
Brannan et al., 2019 [66]	Phase I	Placebo-controlled, N = 69 healthy volunteers, MAD study. Drug exposure: 2-day titration period of either placebo or a KarXT dose of 50 mg xanomeline + 20 mg trospium followed by a 5-day treatment period. The doses (all b.i.d) assessed were xanomeline 100 mg, 125 mg, and 150 mg in combination with trospium 20 mg or 40 mg.	Most cholinergic AEs occurred within the first few days of starting or increasing the study drug. The majority of these AEs at 100 mg and 125 mg xanomeline-dose levels were mild and transient in nature. None of the cohorts showed meaningful changes in orthostatic HR or obvious differences in BP between placebo and KarXT compared to placebo. Increasing trospium dose ameliorated cholinergic AEs and led to the observance of some anticholinergic adverse events (AEs). Some cohorts tested on 40 mg trospium b.i.d reported signs of anticholinergic effects (i.e., dry mouth), particularly in the cohort receiving 125 mg b.i.d of xanomeline.	High
Breier et al., 2023 [78], Kavoussi et al. [66], Karuna Therap. [67]	Phase I, NCT02831231	DBRCT, N = 70 healthy volunteers, xanomeline + placebo or xanomeline + trospium. The dose of xanomeline was 75 mg given three times per day, and the dose of trospium was 20 mg given twice per day. Main outcome: mean weekly maximum composite VAS score (nausea, diarrhea, sweating, salivation, vomiting).	The proportion of subjects reporting any TEAEs was 81.8% on xanomeline alone and 65.7% on KarXT. There was a 46% reduction in the incidence of any cholinergic AEs reported by subjects treated with KarXT compared with xanomeline alone (34.3% vs. 63.6%, respectively). KarXT was associated with a 59% reduction in sweating. In addition, there was a reduction of ≥29% in the incidence of each of the four other individual cholinergic AEs by KarXT compared with xanomeline alone. ECGs, vital signs, and laboratory values were similar between the treatment arms. There were no episodes of syncope in KarXT-treated subjects (two cases occurred in the xanomeline-alone arm), and postural dizziness was noted at lower rates in the KarXT arm (11.4%) compared with xanomeline alone (27.2%).	Moderate

AEs = adverse events; b.i.d = bis in die; CGI-S = Clinical Global Impressions Severity; CGI-I = CGI Improvement; DBRCT = double-blind randomized controlled trial; HR = heart rate; IAQ = Investigator Assessment Questionnaire; KarXT = xanomeline–trospium; MAD = multiple ascending dose; MSQ = Medication Satisfaction Questionnaire; OL = open label; OQD = overall quality of data; PANSS-T = Positive and Negative Syndrome Scale Total; PANSS-P = PANSS positive scale; PANSS-N = PANSS negative scale; PANSS-M-N = PANSS Marder negative scale; TEAEs = treatment-emergent adverse events.

### 3.3. Ongoing and Future Studies

Based on the retrieved data from the US National Institutes of Health, four studies are ongoing, with indications of treatment for schizophrenia and AD. A phase 3, 38-week, randomized, double-blind, placebo-controlled, multicenter outpatient study is expected to enroll 380 patients (aged 55–90) with psychosis associated with AD and has as its primary objective to assess relapse prevention during active intervention vs. placebo [80]. The doses of KarXT used in this trial will be between 20/2 mg t.i.d and 66.7/6.67 t.i.d [80].

An ongoing phase 3, open-label, 56-week study explores the long-term efficacy, safety, and tolerability of KarXT in patients (N = 568) with schizophrenia who receive the investigational product starting from 50/20 mg b.i.d up to 125/30 mg b.i.d [81]. The objectives are assessing the long-term safety and tolerability of KarXT and the characterization of this combination’s pharmacokinetics. The study will monitor the treatment-emergent adverse events (TEAEs), the change in PANSS total, positive, negative, and negative Marder scores, CGI-S scores, and the percentage of responders (30% PANSS total score decrease) at week 52 [81].

An extension phase 3 open-label study is ongoing to explore the long-term efficacy, safety, and tolerability of KarXT in patients with schizophrenia (N = 350) for 53 weeks [82]. A fixed-dose combination of KarXT (125/30 mg b.i.d) is administered, and the main outcomes are the incidence of adverse events, changes in PANSS total score and subscores, CGI-S scores, and response rates [83].

A phase 3, two-part study, with a five-week double-blind phase (randomized, parallel-group, placebo-controlled) and a 12-week open-label extension phase, is ongoing and has as its main objective the evaluation of the safety and efficacy of KarXT in acutely psychotic hospitalized Chinese adults with schizophrenia [84]. The estimated enrollment target is 158 participants aged 18 to 65 years, and the change in total PANSS scores is the main outcome [84].

According to the US National Institutes of Health, four other studies are planned to begin in the near future. A 54-week open-label extension, multicentric, phase 3 roll-over study is intended to assess the long-term safety and tolerability of KarXT in patients with psychosis associated with Alzheimer’s disease (AD) who completed a 38-week study [85]. The estimated enrollment is 140 participants who finished the previously mentioned trial, aged between 55 and 90, and the total daily doses of KarXT (xanomeline–trospium) will be from 20/2 mg up to 200/20 mg [85]. The main outcome measures are the incidence of TEAEs during the entire monitoring period [85].

A 6-week, phase 3 randomized, double-blind, placebo-controlled trial will enroll 400 patients with schizophrenia and inadequate response to their current antipsychotic, and KarXT (50/20 mg b.i.d, up to 125/30 mg b.i.d) will be added to the ongoing treatment [82]. The main outcome of this trial is the change in PANSS total score at week 6; the second outcomes are changes in Personal Social Performance (PSP), CGI-S, and PANSS Marder positive symptom and negative symptom factor score at week 6, “categorical response” rate (≥30% improvement in PANSS total score) at week 6, and “preference of medication” (POM) at week 6 [82].

A phase 3, multicenter, 52-week open-label extension of the previous trial is intended to evaluate the long-term safety and tolerability of KarXT (50/20 mg b.i.d up to 125/30 mg b.i.d) as an add-on in patients with schizophrenia with an inadequate response to the current antipsychotic [86]. The main outcome is the incidence of treatment-emergent adverse events, and the secondary outcomes are the severe adverse events and adverse events leading to drug discontinuation in the estimated 280 patients enrolled in this trial [86].

**Table 4:** Ongoing or planned clinical trials exploring the efficacy, tolerability, and/or safety of KarXT.

Reference	Clinical Trial Phase and Registration Number	Design
Karuna Therap. [80]	Phase 3, NCT05511363 (ADEPT-1)	DBRCT, N = 380 patients with AD + psychosis. Outcomes: relapse prevention with KarXT (20/2 mg t.i.d and 66.7/6.67 t.i.d) vs. placebo during 38 weeks.
Karuna Therap. [81]	Phase 3, NCT04820309 (EMERGENT-5)	OL, N = 568 patients with schizophrenia, KarXT (50/20 mg b.i.d up to 125/30 mg b.i.d), 56 weeks. Outcomes: long-term safety and tolerability of KarXT and description of PK parameters.
Karuna Therap. [83]	Phase 3, NCT04659174 (EMERGENT-4)	Extension phase, OL, N = 350 patients with schizophrenia, 53 weeks, fixed dose of KarXT (125/30 mg b.i.d). Outcomes: PANSS-T, PANSS subscores, CGI-S scores, and response rates.
Karuna Therap. [84]	Phase 3, NCT05919823 (UNITE-001)	DBRCT phase, 5 weeks + OL extension phase, 12 weeks, N = 158 Chinese patients with schizophrenia. Main outcome: PANSS-T.
Karuna Therap. [85]	Phase 3, NCT05980949 (ADEPT-3)	OL, roll-over study, 54 weeks. N = 140 patients with AD + psychosis, KarXT 20/2 mg, up to 200/20 mg/day. Outcome: TEAE incidence.
Karuna Therap. [82]	Phase 3, NCT05145413 (ARISE)	DBRCT, N = 400 patients with schizophrenia and inadequate response to their current antipsychotic, KarXT (50/20 mg b.i.d, up to 125/30 mg b.i.d) + ongoing treatment, 6 weeks. Outcome: PANSS-T, PSP, CGI-S, PANSS-M-P, POM, and response rate (PANSS-T).
Karuna Therap. [86]	Phase 3, NCT05304767	OL extension, 52 weeks, N = 280 patients with schizophrenia and inadequate response to the ongoing antipsychotic, KarXT (50/20 mg b.i.d up to 125/30 mg b.i.d).
Karuna Therap. [87]	Phase 3, NCT06126224 (ADEPT-2)	DBRCT, N = 400 female patients with mild or moderate psychosis associated with AD, KarXT dose of 60/6 to 200/20 mg/day. Outcome: Hallucinations and Delusions score.

AD = Alzheimer’s disease; b.i.d = bis in die; CGI-S = Clinical Global Impressions Severity; DBRCT = double-blind randomized controlled trial; KarXT = xanomeline–trospium; OL = open label; PANSS-M-P = PANSS Marder positive scale; PANSS-T = Positive and Negative Syndrome Scale Total; PK = pharmacokinetics; POM = preference of medication; PSP = Personal Social Performance; TEAEs = treatment-emergent adverse events.

A phase 3, randomized, double-blind, placebo-controlled, parallel-group study is intended to evaluate the safety and efficacy of KarXT in male and female subjects aged 55 to 90 who have mild to severe AD with moderate to severe psychosis related to AD, measuring the Hallucinations and Delusions (H + D) score. The expected number of participants is 400, and the total daily dose of KarXT is between 60/6 mg and 200/20 mg [87].

## 4. Discussion

A total of 14 trials investigating the efficacy, tolerability, safety, and/or pharmacokinetics of xanomeline–trospium were identified through this literature review, and the data were of good quality. These clinical trials were phase I, II, and III, and most of them have not yet published results or are ongoing. Therefore, the discussion about the main parameters of the efficacy and tolerability of KarXT is limited to the results of four trials (two phase I, one phase II, and one phase III). The efficacy of xanomeline–trospium is supported by the changes in the PANSS positive and negative scores, CGI-S scores, five Marder factors on PANSS, and percentage of responders on CGI-S after five weeks of treatment vs. placebo [57,65,73,75].

It is worth mentioning that xanomeline, independent of its association with trospium, was explored in a pilot phase 3 study (N = 20 patients with schizophrenia), and the changes in total PANSS and total BPRS scores supported the significant superiority of the active drug vs. the placebo at week 4 [88]. Also, improvements in cognitive functioning were superior vs. the baseline in patients treated with xanomeline on a cognitive test battery, especially the performances in verbal learning and short-term memory function [88]. The level of significance, when active and placebo groups were compared, was reached by the results of list learning, story recall, delayed memory, and digit span tests [88]. In this pilot study, none of the participants discontinued due to adverse events; however, gastrointestinal adverse events were present more in the active treatment group vs. the placebo, although they were of mild or moderate severity and did not persist for long periods of time; no changes in the Simpson–Angus Rating Scale (SARS), Barnes Rating Scale for Drug-Induced Akathisia (BARS), or Abnormal Involuntary Movement Scale (AIMS) were reported [88]. The presence of gastrointestinal cholinergic adverse events supports the association of trospium to xanomeline.

From the efficacy analysis perspective, the NNT values for KarXT, based on the reviewed data, were between 3 (≥20% improvement in PANSS) and 11 (≥50% improvement in PANSS) [73]. In medicine, an NNT value of 2–5 for monotherapies is considered useful, but this interpretation of this value also depends on the severity of the illness [89,90,91,92]. The role of NNT is to help clinicians compare the clinical efficacy between different interventions, as it measures the effect size [89]. For example, the NNT values for olanzapine were calculated as 8–11, dependent on the active comparators, in patients with schizophrenia, while the NNT for aripiprazole was 6 and for quetiapine was 4 in patients with bipolar disorder, and for risperidone, the NNT values were 6–15 in patients with AD [89,90,91,92].

KarXT led to a significantly superior response vs. the placebo as early as in two weeks [73]. There was a tendency towards more significant enhancement in cognitive function with KarXT compared to the placebo throughout the 5-week treatment period, although statistical significance was not attained [74]. The fact that xanomeline improved cognitive functioning when administered alone in the above-mentioned pilot study supports its recommendation that SSDs associate cognitive impairments in patients with AD with psychosis.

Regarding the tolerability and safety of KarXT, adverse events due to peripheric muscarinic receptor agonism, such as nausea, vomiting, diarrhea, sweating, and hypersalivation, have been reported, raising significant tolerability challenges for case management [57,72]. According to the phase 1 pilot study results, adding trospium to xanomeline led to a 50% decrease in the incidence of muscarinic adverse events compared to xanomeline as monotherapy [77]. As mentioned in Section 3.2., the NNH values for KarXT were between 9 and 23 for pro-cholinergic and between 8 and 13 for anticholinergic adverse effects [71].

Although the role of xanomeline–trospium in the armamentarium of antipsychotics is not yet established because few data are yet available and conducted trials were short-term, there is another important role of the reviewed trials dedicated to this drug combination, i.e., the enhancement of the researchers’ interest in new pathophysiological mechanisms of SSDs. This theoretical direction of investigation supported the appearance of several reviews exploring non-dopaminergic agents for schizophrenia. One of the most recent reviews dedicated to the multidimensional analysis of schizophrenia (definition, etiopathogenesis, clinical expression, therapeutic interventions), conducted by Tandon et al. (2024) [93], highlights that this illness is a heterogenous syndrome and “a single, common pathophysiological pathway appears unlikely”. Another review, conducted by Davidson and Carpenter Jr (2024) [94], acknowledges the fact that currently available therapeutic options for schizophrenia are based on drugs that interfere with dopamine neurotransmission. Until the promises of individualized treatment in patients with SSDs, offered by the genome-wide association studies (GWASs) [95,96], are confirmed, it is necessary to find out more about the neurobiology of these disorders, in order to target them adequately with pharmacological agents.

The review of studies with xanomeline–trospium conducted by Kidambi et al., published in 2023 [50], did not include phase III trials but highlighted the value of early research, both on a practical level and on a more theoretical one, i.e., supporting the role of modulating the cholinergic system in the treatment of psychoses. A more recent review, conducted by Leber et al. in 2024, included four clinical trials and three preclinical studies but did not include a systematic search of the ongoing and future research directions for KarXT [97]. Another review was published in Dutch by Spoelstra et al., in 2023 [98], and included nine published articles on five clinical studies; however, this review explored muscarinic M1 and/or M4 receptor agonists, partial agonists, and positive allosteric modulators, and therefore included not just KarXT and, again, did not include further directions on research in this field. Other reviews dedicated to new antipsychotic agents did not detail KarXT efficacy and tolerability, but they still concluded the need to develop new drugs that support the non-dopaminergic pathogenetic mechanisms of SSDs [25,99,100,101,102].

The limitations of the current review are that (1) it does not include preclinical data, but this was premeditated, as there is at least one review approaching animal studies with xanomeline [97], and the purpose was to find data on the efficacy and tolerability of KarXT in humans; (2) the conclusions are inherently limited to the few trials with available results, and these conclusions may change in the near future, as new results will be posted; and (3) only one clinical trial database was searched for materials regarding KarXT, and only papers published in English were selected.

Future directions of research refer to the need to explore the efficacy and tolerability of xanomeline–trospium in more phase III clinical trials in order to find out the most appropriate dosing regimen for controlling the positive, negative, and cognitive symptoms of schizophrenia, the potential adverse events (especially cholinergic effects), the most adequate ways to mitigate them, etc. Longer duration trials are needed to evaluate the impact of this drug combination on relapse prevention, as the available results refer only to the acute phase of schizophrenia. Also, exploring KarXT as an add-on agent to the ongoing antipsychotic treatment for TRS may be an important direction for future research.

## 5. Conclusions

The association of xanomeline, an agonist of the muscarinic M1 and M4 receptors, and trospium, a peripheral cholinergic antagonist, is a promising treatment for schizophrenia, but this conclusion is based on the results of a very limited number of trials. After reviewing the available data, it can be concluded that (1) the results of four clinical trials support the efficacy of xanomeline–trospium on the positive, negative, and cognitive symptoms of schizophrenia. (2) There are some worries related to the cholinergic adverse events (pro-cholinergic, mainly nausea and vomiting, or anticholinergic, primarily dry mouth and constipation) that may indicate the need to find the most appropriate rapport between the two components of KarXT; in this direction, while the data strongly support the better tolerability for xanomeline–trospium vs. xanomeline monotherapy [66,77,78], the exact ratio between the two components is still debated; the majority of pro-cholinergic adverse events appeared at 100 mg and 125 mg xanomeline, although mild and transient in nature, and increasing the trospium dose ameliorated these adverse events but also led to the observance of some anticholinergic adverse events (at 40 mg trospium b.i.d dose) [66]. (3) There is no head-to-head comparison between KarXT and other antipsychotics, which is normal in this phase of clinical research; therefore, it is too soon to speculate on the superiority or inferiority of non-dopaminergic-based antipsychotics vs. already-in-use dopamine-based antipsychotic agents. (4) Nevertheless, the preliminary data on KarXT suggest that the non-dopaminergic pathogenetic mechanisms of SSDs are worthy of further exploration.

## Figures and Tables

**Table 1:** Inclusion and exclusion criteria.

Operational Criteria	Inclusion Criteria	Exclusion Criteria
Population	All populations were allowed, regardless of the participants’ age (no inferior or superior age limit was pre-defined). The primary diagnoses allowed were schizophrenia and schizoaffective disorder, but patients with all types of severe mental disorders were included in the review. Chronic organic or psychiatric co-morbidities were allowed if screened for and managed adequately, as specified by the trial protocol. Diagnoses should be based on clearly defined criteria, according to ICD10, ICD-11, DSM IV-TR, DSM 5, or DSM 5-TR. No limitation on the initial severity of the disorder (as assessed by a validated scale) was imposed.	Trials that did not specify the demographic and clinical characteristics of the participants. The presence of psychiatric co-morbidities with a significant impact on cognition, mood, and behavior if they were not managed during the trial, based on the specific protocols.
Intervention	Pharmacological intervention with xanomeline–trospium, either as monotherapy or as an add-on. No limitations regarding the dose, way of administration, or duration of the intervention were applied.	Concomitant medication that was not monitored according to the study protocol.
Environment	Both inpatient and outpatient regimen.	Unspecified environment.
Primary and secondary variables	Evaluation of the efficacy, safety, and/or tolerability of xanomeline–trospium.	All research that was using unclear outcomes.
Study design	Any phase of clinical investigation, from I to III, which was focused on evaluating the effects of xanomeline–trospium was admitted.	Studies with unspecified or poorly defined design (e.g., insufficiently validated instruments for monitoring symptom severity, unclear reporting procedures for adverse events, and unspecified study duration). Studies focused on the evaluation of other pharmacological agents as a primary intervention. Case reports, case series, reviews, or meta-analyses. Preclinical studies.

**Table 2:** Risk of bias assessment.

Source Reviewed	RCTs					
	Allocation Concealment	Randomization	Blinding	Outcome Data	Selective Reporting	Others
[65]						
[57]						
[66]						
[67]						

			
No risk of bias was identified		Uncertain risk of bias	The risk of bias is present		Not applicable to that research	

Note: the evaluation of these studies did not find “present risks of bias” or categories of assessment “not applicable to this research”.

## Data Availability

No new data were created; this review processed already published articles.

## Supplementary Materials

The following supporting information can be downloaded at: https://www.mdpi.com/article/10.3390/ph17050610/s1. Table S1. The Preferred Reporting Items for Systematic Reviews and Meta-Analyses (PRISMA) 2020 statement [62]. Table S2. The overall quality of data assessment according to the GRADE recommendations [64].
